# The Human Interferon-Induced MxA Protein Inhibits Early Stages of Influenza A Virus Infection by Retaining the Incoming Viral Genome in the Cytoplasm

**DOI:** 10.1128/JVI.02220-13

**Published:** 2013-12

**Authors:** Han Xiao, Marian J. Killip, Peter Staeheli, Richard E. Randall, David Jackson

**Affiliations:** Biomolecular Sciences Research Complex, University of St Andrews, North Haugh, St Andrews, United Kingdoma; Institute for Virology, University Medical Center Freiburg, Freiburg, Germanyb

## Abstract

The induction of an interferon-induced antiviral state is a powerful cellular response against viral infection that limits viral spread. Here, we show that a preexisting antiviral state inhibits the replication of influenza A viruses in human A549 cells by preventing transport of the viral genome to the nucleus and that the interferon-induced MxA protein is necessary but not sufficient for this process. This represents a previously unreported antiviral function of MxA against influenza A virus infection.

## TEXT

The production of interferons (IFNs), a group of cellular cytokines produced in response to infection by invading pathogens, is an extremely powerful component of the host innate immune response that forms the first line of defense against viral infection. IFNs secreted from virus-infected cells exert their antiviral effects by binding to specific receptors on the surface of uninfected cells. Activated IFN signaling pathways upregulate the production of hundreds of interferon-stimulated genes (ISGs), many of which encode proteins that exhibit antiviral activities, thus creating an antiviral state within these cells (reviewed in reference 1). Viruses have evolved mechanisms to combat the antiviral effects of IFN by encoding proteins with IFN-antagonistic activities. However, these IFN-antagonistic proteins are rarely 100% efficient, as has been shown for a number of viruses, including influenza A virus (2). Therefore, during virus infection, IFN is likely secreted from some virus-infected cells, which induces an antiviral state in the neighboring uninfected cells.

**Fig 1:**
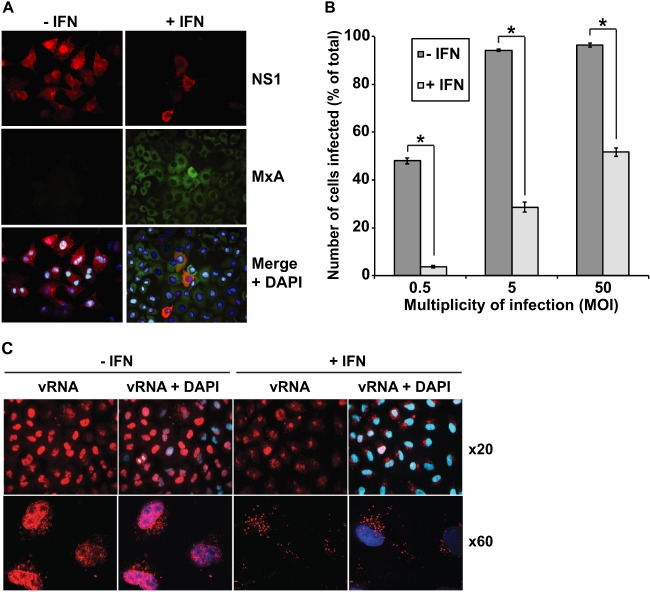
The effects of IFN treatment on viral replication in A/Udorn/72 virus-infected cells. (A) A549 cells were either left untreated or treated with 1,000 U/ml IFN-α for 16 h and subsequently infected with Udorn virus at an MOI of 5 for 12 h. Cells were then fixed in 5% formaldehyde and permeabilized, and immunofluorescence assay performed to detect expression of the viral NS1 protein using an NS1-specific polyclonal antiserum and a Texas red-conjugated secondary antibody (red) or the MxA protein using a specific polyclonal antiserum (Santa Cruz Biotechnology, USA) and a fluorescein isothiocyanate (FITC)-conjugated secondary antibody (green). Cell nuclei were detected using 4′,6′-diamidino-2-phenylindole (DAPI) (blue). Images were taken at ×40 magnification using a Nikon Microphot-FXA fluorescence microscope and overlaid using Adobe Photoshop CS5 software. (B) A549 cells were treated with 1,000 U/ml IFN-α for 16 h and subsequently infected with Udorn virus at the indicated MOI for 12 h. Cells were then subjected to flow cytometry analysis in which virus-infected cells were detected using the anti-NS1 antibody and a phycoerythrin-conjugated secondary antibody. Ten thousand cells were analyzed using a BD FACScan flow cytometer, and data were subsequently analyzed using FlowJo (Treestar). Results are expressed as the averages of three independent experiments ± standard deviations. Statistical significance was assessed by Student's *t* test (*, *P* < 0.0001). (C) Virus input assay of viral genome distribution by fluorescence *in situ* hybridization (FISH). A549 cells were treated with 1,000 U/ml IFN-α for 16 h and either mock infected or infected with WSN virus at an MOI of 500 in the presence of 100 μg/ml cycloheximide (CHX) at 4°C for 30 min. Cells were incubated at 37°C for 2 h in the presence of CHX, fixed, and stained via FISH using a DIG-labeled probe (DIG RNA labeling kit; Roche, United Kingdom) specific for vRNA segment 8 (vRNA; red). Cell nuclei were detected using DAPI (blue). Images were taken at ×20 and ×60 magnification using a DeltaVision RT deconvoluting microscope (Applied Precision).

To observe the effects of an IFN-induced antiviral state on influenza virus infection, human lung epithelial A549 cells untreated or pretreated with 1,000 U/ml alpha interferon (IFN-α) for 16 h were infected with A/Udorn/72 (H3N2) (Udorn) virus at a multiplicity of infection (MOI) of 5. Cells were fixed at 12 h postinfection (h.p.i.) and probed for the expression of both the viral NS1 protein and MxA by immunofluorescence assay (Fig. 1A). MxA is an IFN-induced protein that is regulated tightly only by type I or type III IFN signaling (3), and thus, it is often used as a marker for the IFN-induced antiviral state. The results in Fig. 1A show that, while virtually all non-IFN-treated cells were positive for viral antigen, viral replication occurred only in a minority of IFN-treated cells. However, these cells were also positive for MxA, suggesting that viral replication was able to overcome the antiviral state in this minority of cells. Samples fixed at 48 h.p.i. contained numbers of antigen-positive cells similar to those fixed at 12 h.p.i, suggesting that, in the majority of cells, the infecting virus was never able to overcome the IFN-induced block in viral replication (data not shown).

To determine whether this IFN-mediated antiviral effect could be overcome by increasing the amount of input virus, IFN-treated or untreated A549 cells were infected with Udorn virus at a range of increasing MOIs, fixed at 12 h.p.i., and probed for the expression of NS1 by flow cytometry (Fig. 1B). The degree of protection by IFN gradually decreased with increasing MOI, although even at an MOI of 50, only 52% of IFN-treated cells became positive for viral antigen. Under these experimental conditions, 97% of untreated A549 cells became virus antigen positive. These results indicate that the degree of IFN-mediated inhibition of virus replication depends on the multiplicity of infection and that influenza virus is able to overcome the IFN-mediated antiviral state by swamping the cell with high numbers of infectious virus particles.

To ensure that the above-described findings were not a result of a virus strain-dependent effect, all experiments were repeated using the A/WSN/33 (H1N1) (WSN) strain, and all results were virtually identical to those described above (data not shown).

The initial stages of viral entry after IFN treatment were analyzed using fluorescent *in situ* hybridization (FISH) in a virus input assay to monitor the cellular location of the viral genome from the initiation of virus-induced endocytosis through to nuclear import. To generate sufficient signal for detection by FISH, the initial infection required an MOI of 500, which required the use of the WSN virus strain. IFN-treated or untreated A549 cells were inoculated with WSN virus at 4°C to synchronize the infection and in the presence of cycloheximide (CHX) to ensure that only input viral genomes were detected, due to the global inhibition of protein synthesis and the prevention of viral replication. At 30 min after inoculation, the cells were warmed to 37°C to initiate endocytosis, and at 2 h.p.i., FISH was performed using a digoxigenin (DIG)-labeled probe that hybridized to viral genomic RNA (vRNA) segment 8. vRNA predominantly localized inside the cell nuclei in the absence of IFN treatment; however, in the majority of IFN-treated cells, vRNA localized outside the nucleus at the perinuclear region (Fig. 1C). Therefore, pretreatment of cells with IFN-α inhibits subsequent influenza virus infection at or immediately prior to the stage of nuclear vRNA import.

**Fig 2:**
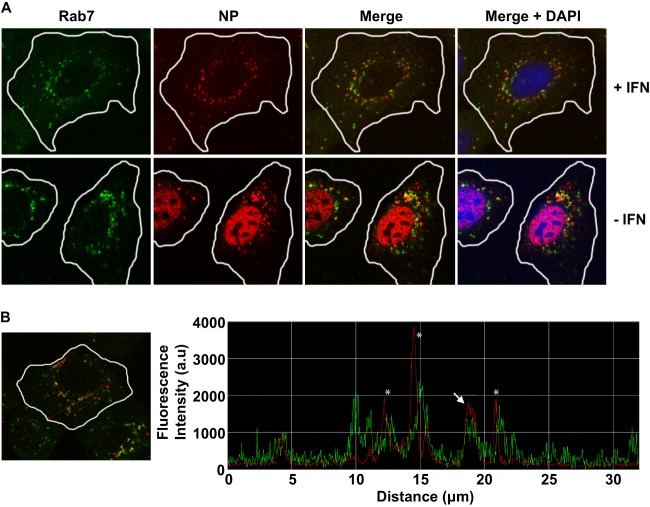
IFN treatment results in the retention of viral genome either inside or in the immediate vicinity of late endosomes. (A) Virus input assay. Naive A549 cells were either left untreated or treated with 1,000 U/ml IFN-α for 16 h and subsequently infected with WSN virus at an MOI of 500 in the presence of 100 μg/ml CHX at 4°C for 30 min. Cells were then incubated at 37°C for 2 h in the presence of CHX, fixed in 5% formaldehyde, permeabilized, and subjected to immunofluorescence analysis. The cellular distribution of NP (red) was determined using an NP-specific monoclonal antibody (Abcam, United Kingdom), and the expression of the late endosomal marker Rab7 (green) was analyzed using a Rab7-specific monoclonal antiserum (Abcam, United Kingdom). Cellular nuclei were visualized using DAPI (blue). Images were taken at ×63 magnification using a Zeiss Pascal 510 confocal microscope and LSM 5 Exciter software. Images were processed using LSM 5 Image Examiner software (Zeiss). The plasma membrane is outlined in white based on the differential interference contrast (DIC) image of the selected cells; the outline was overlaid onto the images using Photoshop CS5 software. (B) The distribution of high levels of NP (red) and Rab7 (green) fluorescence intensity through various planes of infected cells was determined using LSM 5 Image Examiner software. An example of this analysis is shown. The fluorescence intensities of NP (red) and Rab7 (green) along the red line through the selected cell are plotted in the histogram. The distance covered along the red line is indicated on the *x* axis. Peaks of NP and Rab7 that are immediately adjacent to each other are highlighted by asterisks, and a peak of colocalization is highlighted by the white arrow. a.u, arbitrary units.

The punctate distribution of vRNA in Fig. 1C suggests that after IFN pretreatment, the incoming viral ribonucleoprotein complexes (vRNPs) may be retained in late endosomes. To address this, naive A549 cells were either treated with IFN or left untreated for 16 h and subjected to a virus input assay in the presence of CHX, similar to that described for the FISH experiments except that vRNP localization was detected by immunofluorescence using an NP-specific monoclonal antibody. Late endosomes were identified using a rabbit anti-Rab7 monoclonal antibody. Figure 2A shows colocalization of NP and Rab7 for only a minority of input genomes; however, all areas of punctate NP staining are immediately adjacent to a late endosome, as determined by Rab7 staining. This was confirmed by measuring the fluorescence intensity and distance between areas of NP and Rab7 staining in various planes through the cell. An example of such analysis shows that, in a minority of cases, NP and Rab7 overlap (Fig. 2B, white arrow), which may indicate viral genome trapped inside the late endosome. In the majority of cases, the areas of intense NP and Rab7 staining did not overlap (Fig. 2B, asterisks); however, all areas of intense NP staining were found within 1 μm of Rab7 staining. This may suggest that the viral genome has been released from the late endosome but its transport to the nucleus is inhibited by one or more ISG.

**Fig 3:**
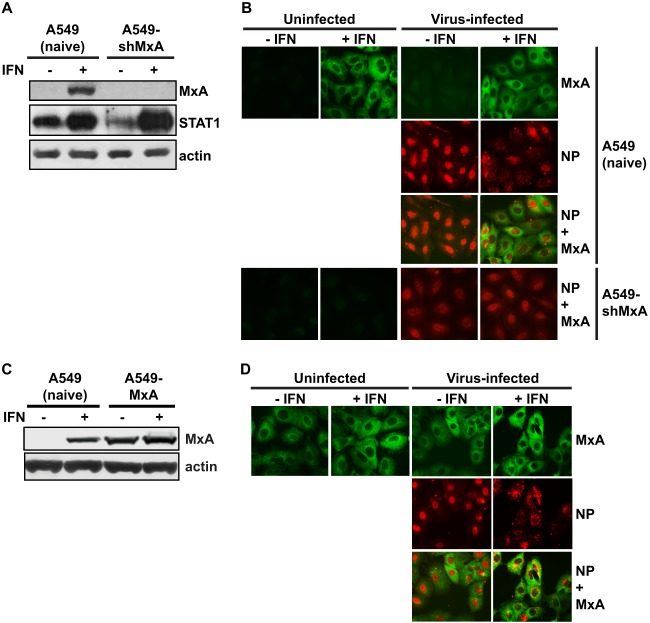
The effects of MxA depletion and overexpression on nuclear import of viral genomic RNA. (A) Naive A549 and A549-shMxA cells were either left untreated or treated with 1,000 U/ml IFN-α for 16 h. Cells were lysed, and protein content was analyzed by SDS-PAGE and immunoblotting using primary antibodies specific for MxA and STAT1. β-Actin was analyzed as a loading control. (B) Virus input assay. Naive A549 and A549-shMxA cells were either left untreated or treated with 1,000 U/ml IFN-α for 16 h and subsequently mock infected (uninfected) or infected with WSN virus at an MOI of 500 in the presence of 100 μg/ml CHX at 4°C for 30 min. Cells were then incubated at 37°C for 2 h in the presence of CHX, fixed in 5% formaldehyde, permeabilized, and subjected to immunofluorescence analysis. The cellular distribution of NP (red) was determined using an NP-specific monoclonal antibody (Abcam, United Kingdom), and the expression of MxA (green) was analyzed using the MxA-specific polyclonal antiserum. Images were taken at ×40 magnification using a Nikon Microphot-FXA fluorescence microscope and overlaid using Adobe Photoshop CS5 software. (C) Naive A549 and A549-MxA cells were either left untreated or treated with 1,000 U/ml IFN-α for 16 h. Cells were lysed, and protein content was analyzed by SDS-PAGE and immunoblotting using the MxA-specific polyclonal antiserum. β-Actin was analyzed as a loading control. (D) A549-MxA cells were used in a virus input assay as described for panel B.

To characterize this block in viral replication, the specific ISGs responsible must be identified. One potential candidate is the IFN-induced MxA protein, which has been shown to have antiviral activity against a number of DNA and RNA viruses, many of which are specifically inhibited via MxA interacting with the viral nucleocapsid proteins (reviewed in reference 4). Previous reports suggest that MxA has antiviral activity against influenza A virus (reviewed in references 4 and 5) and that MxA specifically targets influenza virus nucleoprotein (NP) (6, 7), although the exact mechanism of this antiviral activity is unknown. To determine whether MxA plays a role in preventing transport of the viral genome to the nucleus, an MxA knockdown A549 cell line (A549-shMxA) was generated using a lentivirus small hairpin RNA (shRNA) expression system. Immunoblot and immunofluorescence analysis were used to determine the extent of MxA knockdown in A549-shMxA cells (Fig. 3A and B). Naive A549 cells and A549-shMxA cells were treated with 1,000 U/ml IFN-α for 16 h, and cells were either lysed for immunoblot assay or fixed for immunofluorescence assay with a monoclonal anti-MxA antibody. In contrast to naive A549 cells, MxA could not be detected in A549-shMxA cells (Fig. 3A and B); however, the expression of STAT1 was induced, thereby confirming that A549-shMxA cells were still able to respond to IFN (Fig. 3A).

Naive A549 or A549-shMxA cells were either treated with IFN or left untreated for 16 h and subjected to a virus input assay in the presence of CHX. In untreated A549-shMxA cells, NP was predominantly observed in the nucleus (Fig. 3B). However, in contrast to naive A549 cells after IFN treatment, the vRNPs were efficiently imported into the nucleus of A549-shMxA cells. Therefore, depletion of MxA prevented the IFN-mediated retention of viral genome outside the nucleus.

To determine whether MxA expression alone is sufficient for this effect, an MxA-overexpressing cell line (A549-MxA) was generated and the expression of MxA was determined by immunoblot and immunofluorescence assays (Fig. 3C and D). MxA accumulated in the cytoplasm of A549-MxA cells with a granular staining pattern similar to that observed in naive A549 cells treated with IFN (Fig. 3B). A virus input assay demonstrated that the distribution of vRNPs in both untreated and IFN-treated A549-MxA cells was similar to that in naive A549 cells, suggesting that MxA overexpression alone is not sufficient to prevent the transport of the viral genome to the nucleus (Fig. 3D).

**Fig 4:**
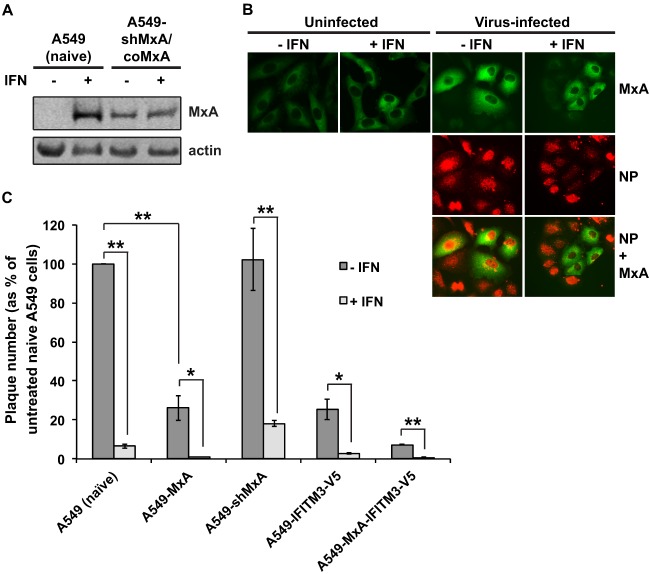
Overexpression of coMxA in MxA-depleted cells restores the block in vRNP nuclear import but only after IFN treatment. (A) Naive A549 and A549-shMxA/coMxA cells were either left untreated or treated with 1,000 U/ml IFN-α for 16 h. Cells were lysed, and protein content was analyzed by SDS-PAGE and immunoblotting using MxA-specific polyclonal antiserum. β-Actin was analyzed as a loading control. (B) A549-shMxA/coMxA cells were used in a virus input assay as described in the legend to Fig. 3B. (C) Naive A549, A549-MxA, A549-shMxA, A549-IFITM3-V5 (A549 cells stably expressing V5-tagged IFITM3 created by lentivirus expression), and A549-MxA-IFITM3-V5 cells (expressing both MxA and V5-tagged IFITM3) cells were either left untreated or treated with 1,000 U/ml IFN-α for 16 h. The cells were then inoculated with a serial 10-fold dilution series of Udorn virus in a viral plaque assay as previously described (16). The cells were fixed 5 days postinfection and immunostained using an anti-X31 polyclonal antibody (16), and plaque number was determined. Plaque number is expressed as the percentage of plaques observed on untreated naive A549 cells. Results are expressed as the average of three independent experiments ± standard deviation. Statistical significance was assessed by Student's *t* test (*, *P* = 0.002; **, *P* < 0.001).

It was possible that the exogenously expressed MxA protein in A549-MxA cells was nonfunctional. To address this issue, a stable cell line was created in which codon-optimized MxA (coMxA) was introduced into A549-shMxA cells (A549-shMxA/coMxA cells). The nucleotide sequence of the coMxA gene contains multiple synonymous substitutions such that no shRNA-complementary sequences are present. Due to the shRNA-mediated knockdown of endogenous MxA, only coMxA is expressed in A549-shMxA/coMxA cells either with or without IFN treatment (Fig. 4A and B). In a virus input assay, incoming vRNPs were localized in the nucleus of all A549-shMxA/coMxA cells without IFN treatment, regardless of the expression level of coMxA. After IFN treatment, vRNPs were localized in the nucleus of cells displaying low or undetectable levels of coMxA. However, in cells expressing high levels of coMxA, the vRNPs were predominantly localized to the cytoplasm (Fig. 4B), indicating that the ability of IFN to block the transport of influenza virus vRNPs to the nucleus was restored by reintroducing MxA into MxA knockdown cells.

Despite the fact that MxA expression is required to prevent vRNP transport to the nucleus, overexpression of MxA alone is insufficient to account for the observed block. It is possible that a form of IFN-induced modification of MxA is required for its antiviral activity. However, a more likely explanation is that there are other IFN-induced auxiliary molecules that act in conjunction with MxA to restrict vRNP nuclear import, which is implied by the data shown in Fig. 4C. Udorn virus was titrated by standard plaque assay in untreated or IFN-pretreated cells, and plaque numbers were quantified at 4 days postinfection (Fig. 4C). Compared to the results for untreated naive cells, there was a 74% reduction in the number of plaques in untreated MxA-expressing cells, suggesting that MxA exhibited antiviral activity in the absence of IFN treatment. However, the plaque number was still 4-fold higher in untreated MxA-expressing cells than in IFN-treated naive cells. IFN treatment of naive cells resulted in a 15-fold reduction in plaque number, whereas in MxA knockout cells, there was only a 5-fold reduction in plaque number after IFN treatment. However, the plaque number was only 18% that of untreated naive cells, suggesting that the plaque reduction in IFN-treated A549-shMxA cells was as a result of at least one ISG product other than MxA. These findings suggest that at least one other ISG product is required alongside MxA to cause the inhibitory effect on influenza virus replication observed after IFN treatment.

The cellular location and appearance of the input viral genome after IFN treatment is suggestive of location in or in the immediate vicinity of the endosome in which the virus entered the cell. A similar phenomenon has been reported upon the expression of IFN-inducible transmembrane protein 3 (IFITM3) (8), a protein with known antiviral activity against influenza A viruses (8–10). IFITM3 expression restricts input viral genome to the endosome by preventing viral fusion, and it was therefore suggested that IFITM3 is necessary and sufficient for blocking the nuclear import of influenza vRNPs (8). However, our data suggest that IFITM3 cannot be sufficient for this effect, as in cells lacking MxA, vRNPs are still efficiently imported into the nucleus after IFN treatment (Fig. 3B). We have observed that the expression of either MxA or IFITM3 has a similar effect on plaque number in A549 cells, and we noted an enhanced antiviral effect when both IFITM3 and MxA are overexpressed (Fig. 4C). We therefore suggest that, although IFITM3 is undeniably important in preventing influenza virus infection, MxA also has a significant and profound effect on preventing transport of the viral genome to the nucleus.

Previous work regarding the antiviral effects of human MxA in a mouse cell line indicated a role of MxA in blocking influenza A virus replication after the stage of primary transcription (11), possibly by interacting with the viral NP and preventing it from entering the nucleus to participate in secondary transcription. This mechanism of MxA action is likely, given that NP has been shown to be the viral target of Mx proteins (7, 12) and a direct interaction between MxA and influenza A virus NP has been previously described (6). However, a recent report in which similar properties of MxA were examined in primate cells described an antiviral effect of MxA prior to the stage of primary transcription, as the levels of all viral RNA species were reduced in infected cells expressing MxA (13). Here, we show that this alternative antiviral effect of MxA at an earlier stage of the viral replication cycle occurs by preventing the input vRNPs from being transported to the nucleus. A similar antiviral effect of MxA has previously been shown against Thogoto virus (14), which suggests that this function of MxA is effective against orthomyxoviruses in general and, potentially, against other viruses that replicate within the nucleus. Interestingly, MxA seems to require one or more additional IFN-induced factors for preventing the endosomal-nuclear transport of the influenza A virus genome but not that of Thogoto virus. Molecular models of the oligomeric MxA protein structure suggest that binding of MxA around vRNPs is entirely possible (15). This adds to the hypothesis that MxA may be able to interact directly with incoming vRNPs in an early stage of viral infection, thereby preventing vRNPs from entering the nucleus. Further work is required to fully characterize how MxA interacts with influenza virus proteins/complexes during the various stages of the viral replication cycle and to elucidate the nature of the putative MxA cofactor.
